# An immune clock of human pregnancy

**DOI:** 10.1126/sciimmunol.aan2946

**Published:** 2017-09-01

**Authors:** Nima Aghaeepour, Edward A. Ganio, David Mcilwain, Amy S. Tsai, Martha Tingle, Sofie Van Gassen, Dyani K. Gaudilliere, Quentin Baca, Leslie McNeil, Robin Okada, Mohammad S. Ghaemi, David Furman, Ronald J. Wong, Virginia D. Winn, Maurice L. Druzin, Yaser Y. El-Sayed, Cecele Quaintance, Ronald Gibbs, Gary L. Darmstadt, Gary M. Shaw, David K. Stevenson, Robert Tibshirani, Garry P. Nolan, David B. Lewis, Martin S. Angst, Brice Gaudilliere

**Affiliations:** 1 Department of Anesthesiology, Perioperative and Pain Medicine, Stanford University School of Medicine, Stanford, CA 94121, USA; 2 Department of Microbiology and Immunology, Stanford University, Stanford, CA 94121, USA; 3 Department of Information Technology, Ghent University, and the Center for Inflammation Research, Ghent, Belgium; 4 Department of Surgery, Stanford University School of Medicine, Stanford, CA 94121, USA; 5 Institute for Immunity, Transplantation and Infection, Stanford University School of Medicine, Stanford, CA 94305, USA; 6 Institute for Immunogenetics, Jose de San Martin Clinical Hospital, National Scientific and Technical Research Council (CONICET), Buenos Aires, Argentina; 7 Department of Pediatrics, Stanford University School of Medicine, Stanford, CA 94121, USA; 8 Department of Obstetrics and Gynecology, Stanford University School of Medicine, Stanford, CA 94121, USA; 9 Departments of Biomedical Data Sciences and Statistics, Stanford University, Stanford, CA 94121, USA; 10 Division of Pediatric Immunology and Allergy, Stanford University School of Medicine, Stanford, CA 94121, USA

## Abstract

Themaintenance of pregnancy relies on finely tuned immune adaptations.We demonstrate that these adaptations are precisely timed, reflecting an immune clock of pregnancy in women delivering at term. Using mass cytometry, the abundance and functional responses of allmajor immune cell subsets were quantified in serial blood samples collected throughout pregnancy. Cell signaling–based Elastic Net, a regularized regressionmethod adapted from the elastic net algorithm, was developed to infer and prospectively validate a predictive model of interrelated immune events that accurately captures the chronology of pregnancy. Model components highlighted existing knowledge and revealed previously unreported biology, including a critical role for the interleukin-2–dependent STAT5ab signaling pathway in modulating T cell function during pregnancy. These findings unravel the precise timing of immunological events occurring during a term pregnancy and provide the analytical framework to identify immunological deviations implicated in pregnancy-related pathologies.

## INTRODUCTION

During pregnancy, the maternal immune system must engage in a fine balancing act: maintaining tolerance to the fetal allograft while preserving innate and adaptive immune mechanisms for protection against microbial challenges (*1*, *2*). The dysregulation of immunological mechanisms normally engaged in the maintenance of a term pregnancy is increasingly implicated in the pathogenesis of preterm birth and other pregnancy-related complications (*2*–*4*). The capacity to capture such dysregulation during the course of pregnancy in an accessible body compartment (such as peripheral blood) is of high clinical importance, because it will enable risk prediction and alleviation. A critical question is whether a chronology of precisely timed immune adaptations, evocative of an “immune clock,” can be tracked in peripheral blood during term pregnancy. Demonstrating that an immune clock characterizes term pregnancy is a key premise to search for chronological deviations associated with pathologies of gestation such as preterm birth.

Elucidating the feto-maternal immunological cross-talk implicated in themaintenance of a termpregnancy has been the subject of substantial interest. Animal and human studies investigating the role of the feto-maternal interface have been particularly useful in revealing the local cellular mechanisms that control maternal immune tolerance to the semi-allogeneic fetus (*1*, *2*, *4*, *5*). In contrast, fewer efforts have focused on the systemic immune adaptations to pregnancy. Seminal studies centered on plasma cytokine profiles and flow cytometry analyses of isolated peripheral leukocytes have provided important clues to the understanding of the peripheral immune response during pregnancy (*6*–*9*). These studies are consistent with gestational age–dependent and cell type–specific immune adaptations, which provide the mother with effective protection fromharmful pathogenswhile allowing for tolerance to fetal antigens (*5*, *10*). For example, some aspects of adaptive immunity are decreased during pregnancy, such as T cell and B cell frequencies and the ability of naïve CD4^+^ T cells to produce T helper cell 1 (T_H_1)– and T_H_2-type cytokines. In contrast, specific innate immune responses are exacerbated, such as natural killer (NK) cell, monocyte, and plasmacytoid dendritic cell (pDC) cytokine responses when stimulated with viral particles (*8*, *11*). However, the limited information at the cellular level afforded by proteomic analyses, the drawbacks of using isolated cells removed from their natural multicellular milieu, and the computational challenges presented by the statistical interpretation of high-dimensional immune networks have thus far precluded the characterization of the chronology underlying these immune system–wide adaptations to pregnancy.

The recent “bedside” applications of high-content immune profiling technologies, including mass cytometry, hold significant promise for providing comprehensive insights into immunological processes implicated in normal and pathological pregnancies (*12*, *13*). Mass cytometry is a single-cell analysis platform that enables the simultaneous interrogation of multiple signaling pathways in distinct cell subsets spanning the entire immune system (*14*). The multiplex functional readout afforded by mass cytometry provides unprecedented opportunities to describe the human immune system as a network of correlated, cell type–specific attributes and to investigate the functional relationships between cells within and across hematopoietic lineages (*15*, *16*). However, as the number of measured parameters increases, the development of appropriate computational methods that make optimal use of the dimensionality and correlated nature of the data while avoiding “overfitting” has become paramount (*17*).

In this study, we combined the high-parameter functional profiling of peripheral immune cells with a previously unknowncell signaling–based Elastic Net (csEN) algorithm to infer a model of interrelated immune features that accurately predicts the timing of immunological adaptations over the entire course of a term pregnancy. Such a model can then serve as a reference to detect immunological disruptions that may precede clinical manifestations of pregnancy-related pathologies. The csEN algorithm was adapted from the ElasticNet (EN) regularized regression method (*18*) and accounts for the influence of previous biological knowledge of receptor-specific signal transduction on the generation of single-cell mass cytometry data. Here, the immunological data set comprised the phenotype and intracellular signaling activities of all major innate and adaptive immune cells, which were simultaneously quantified in serial whole-blood samples. Three sets of data were generated by quantifying the abundances of peripheral immune cell subsets, capturing endogenous intracellular signaling activities, and determining the capacity of immune cell subsets to respond to stimulation with receptor-specific ligands.

## RESULTS

### Deep single-cell profiling of the systemic immune response to pregnancy

Twenty-one pregnant women receiving routine antepartum care at Lucile Packard Children’s Hospital were enrolled in the study. Patient demographics, antepartum parameters, mode of delivery, and gestational age at delivery are listed in Table 1. The analysis was performed on 18 patients who delivered at term (≥37 weeks of gestation). Three women who delivered prematurely (<37 weeks of gestation) were excluded from the analysis.An independent cohort of 10 pregnant women who delivered at term was subsequently enrolled to prospectively validate study results.

**Table 1:** Patient and pregnancy characteristics.

	Training cohort (*n* = 18)	Validation cohort (*n* = 10)
**Demographics**		
Age (years, mean ± SD)	31.9 ± 3.4	32.8 ± 5.1
Body mass index (kg/m^2^, mean ± SD)	23.8 ± 6.5	26.3 ± 2.2
Gravity [median (interquartile range)]	1.5 (1, 7)	2 (1, 5)
Parity (% nulliparous)	50	30
Race (%)		
Caucasian	15 (83)	7 (70)
Others (Asian, African, or American Indian)	2 (11)	3 (30)
Unknown	1 (6)	0
Ethnicity (% Hispanic)	3 (11)	2 (20)
Married (%)	14 (78)	8 (80)
Level of education (%)		
≤High school	2 (11)	1 (10)
>High school	16 (89)	9 (90)
**Current pregnancy parameters**		
Gestational age at delivery (weeks, mean ± SD)	38.9 ± 1.4	39.5 ± 1.3
Mode of delivery (%)		
Spontaneous	13 (72)	5 (50)
Cesarian section	5 (18)	5 (50)
Birth weight (kg, mean ± SD)	3.2 ± 0.4	3.7 ± 0.4
Five-minute Apgar score [media (range)]	9 (8–9)	9 (8–9)

**Fig. 1:**
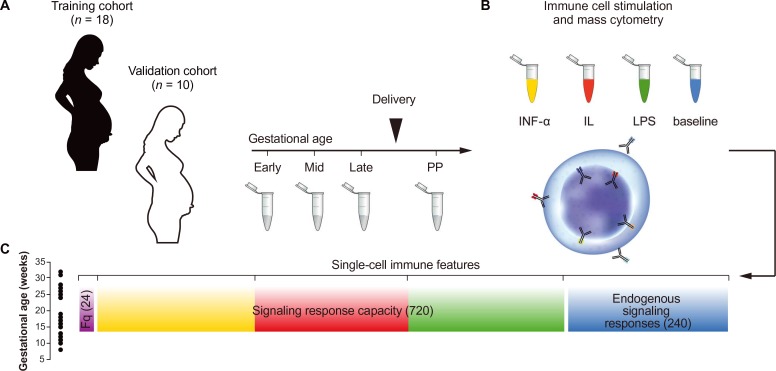
**Experimental workflow and analytical approach.** (**A**) Eighteen women who delivered at term gestation were initially studied. Ten additional women were subsequently enrolled as a validation cohort. A whole-blood sample was obtained at three time points (early, mid, and late) during pregnancy and 6 weeks postpartum (PP). (**B**) Aliquots were either left unstimulated (to quantify cell frequency and endogenous intracellular signaling activity) or stimulated with a panel of receptor-specific ligands, including IFN-α, a cocktail of ILs containing IL-2 and IL-6 (IL), and LPS. Immune cells were stained with surface and intracellular antibodies and analyzed with mass cytometry. (**C**) The assay produced three categories of immune features, providing information about cell frequencies (Fq) measured in 24 manually gated immune cell subsets (purple bar), cell type–specific signaling capacity to respond to exogenous ligands (IFN-α, yellow bar; IL, red bar; LPS, green bar), and endogenous signaling activity (blue bar). The number of immune features contained within each data category is indicated in parentheses. The analyses used the variability in sample collection time to define a continuous variable (gestational age at time of sampling in weeks) distributed across the course of pregnancy (left, black circles).

Serial blood samples were collected at three time points during pregnancy (early-, mid-, and late-pregnancy time points) and at 6 weeks postpartum (Fig. 1A). The approach leveraged the interindividual variability in sample collection time to define a continuous variable (gestational age at time of sampling) distributed across the course of pregnancy. Using a 41-parameter mass cytometry assay (table S1), a total of 984 immune features were extracted from each blood sample including the frequencies of 24 immune cell subsets representing major innate and adaptive compartments (fig. S1), their endogenous intracellular signaling activities, and the capacities of each cell subset to respond to a series of receptor-specific immune challenges [stimulation with lipopolysaccharide (LPS), interferon-α2A (IFN-α), and a cocktail of interleukins (ILs) containing IL-2 and IL-6] (Fig. 1, B and C). Immune features were derived from measurements performed in whole-blood samples, which allowed the functional assessment of all peripheral immune cell subsets while minimizing perturbations by experimental processing.

### An immune clock of pregnancy: csEN predicts the dynamic changes of the maternal immune system over the course of pregnancy

**Fig. 2:**
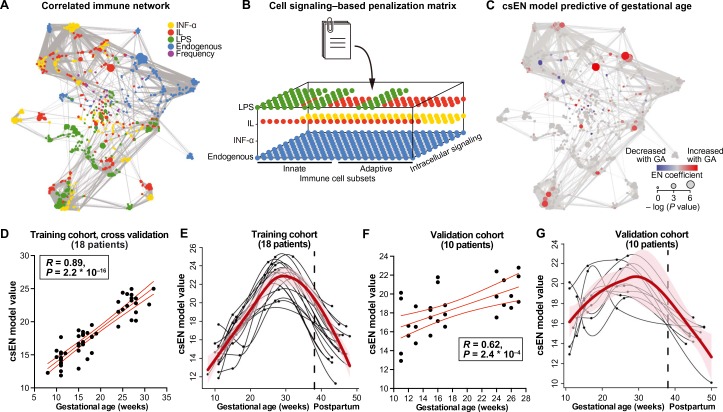
**A prospectively validated csEN model accurately predicts dynamic changes of the maternal immune system over the course of pregnancy.** (**A**) Correlation network revealing the relationships between immune features within and acrossmass cytometry data categories (Spearman’s coefficient). (**B**) Cell signaling–based penalization matrix that allowed prioritizing canonical, receptor-specific signaling responses (see Materials and Methods). Signaling responses amenable to prioritizing are highlighted in blue (endogenous), yellow (IFN-α), red (IL), or green (LPS). (**C**) Cross-validated csEN model predicting gestational age at time of sampling. Red/blue dots highlight model components that trended upward/downward during pregnancy. Dot size indicates the correlation between model component and gestational age (Spearman’s coefficient). (**D** and **E**) Training cohort. (D) csENmodel prediction of the gestational age at time of sampling (*R* = 0.89, *P* = 2.2 × 10^−16^, *n* = 18, cross-validation). (E) Line plots depicting csEN model values for each patient during pregnancy and for the postpartumsamples. Red lines and red shadow representmedian and 95%confidence interval of EN components. (**F** and **G**) Validation cohort. (F) csENmodel prediction of the gestational age at time of sampling (*R*= 0.62, *P*= 2.4× 10^−4^, *n* = 10, Spearman’s coefficient). (G) Line plots depicting csEN model values for each patient (validation cohort).

The mass cytometry data set formed a correlation network emphasizing the interconnectivity of measured immune features (Fig. 2A and the Supplementary Materials). The highly correlated nature of the mass cytometry data suggested an EN method—a regularized regression method particularly suitable to the analysis of intercorrelated data (*18*)—as a primary candidate to determine whether dynamic changes occurring in peripheral immune cells during pregnancy could predict gestational age at time of sampling.

csEN, an extension of the existing EN algorithm, was developed to account for the fact that previous biological knowledge of intracellular signal transduction exerts an inherent influence on the generation of mass cytometry data sets. In our study, ligands for ex vivo stimulation were selected a priori to evoke cell-intrinsic and receptor-specific signaling responses [e.g., Toll-like receptor (TLR) 4–dependent signaling for LPS, Janus kinase (JAK)/signal transducer and activator of transcription (STAT) signaling for IFN-α, and JAK/STAT and mitogen-activated protein kinase (MAPK) for the type I ILs IL-2 and IL-6]. Although these stimulations may activate important intracellular cross-talk and receptorindependent signaling responses, we hypothesized that emphasizing canonical, receptor-specific signaling responses would improve the robustness of the analysis. We implemented a cell signaling–basedmatrix constructed a priori that indicated for each cell subsetwhether a receptorspecific signaling response to each stimulation condition is supported by prior knowledge of intracellular signal transduction (Fig. 2B and table S2). In analogy to L1 and L2 penalties of the EN algorithm, a third penalty function was introduced that allowed, but did not force, prioritizing signaling responses included in the cell signaling–based matrix. The magnitude of such penalization was agnostically and objectively determined, resulting in a 5:1 prioritization of knowledge-based signaling responses (see Materials and Methods).

The csEN algorithm identified a predictive model in the first cohort of pregnant women that was strongly associated with gestational age at the time of sampling (*R* = 0.89, *P* = 2.2 × 10^−16^; Fig. 2, C to E). The validity of the ENmodel was prospectively tested in themass cytometry data from a cohort of 10 additional women. The EN model strongly predicted gestational age at time of sampling in this independent cohort (*R* = 0.62, *P* = 2.4 × 10^−4^; Fig. 2, F and G). Although the analysis only considered data collected during pregnancy, applying the coefficients of the csENmodel to postpartum data allowed for comparison of the maternal immune state during and after pregnancy (Fig. 2, E and G). The csEN model agnostically placed the postpartum state closest to the earliest stage of pregnancy.

**Fig. 3:**
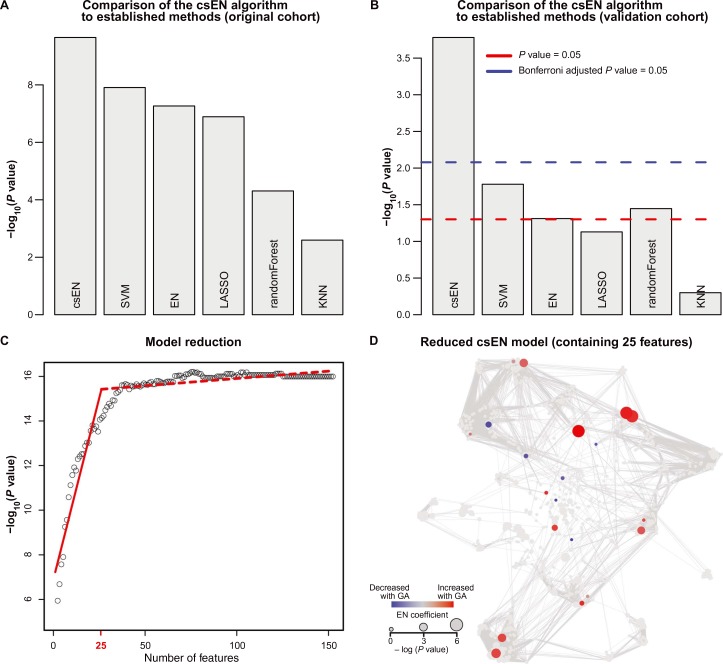
**Comparison of the csEN algorithmto existing predictive methods and model reduction.** (**A** and **B**) Comparison of the predictive power of existing algorithms for the estimation of gestational age at time of sampling in the training cohort (A) (*n* = 18) and the validation cohort (B) (*n* = 10). Algorithms included Support Vector Machine (SVM), EN, LASSO, randomForest, and *k*-nearest neighbors (KNN). (**C**) The dot plot depicts the number of csEN model components versus the *P* value of the csEN model for predicting gestational age. Red lines indicate the piece-wise regression fit for identification of a breakpoint indicating that 25 features are required for highest statistical stringency. (**D**) Location of the 25 features in the correlation network.

Together, the analysis identified a precisely timed chronology of interrelated immune events that differed from a nonpregnant state and progressed dynamically over the course of pregnancy. A side-by-side comparison of the predictive power for estimating the gestational age variable demonstrated that the csEN method significantly improved the existingENalgorithmand outperformed algorithms representing the most common alternative predictive methods including randomForest (*19*), *k*-nearest neighbors (*20*), Support Vector Machines (*21*), and LASSO (Fig. 3, A and B) (*22*). In comparison with the less predictive, non–signaling-based EN model, the csEN algorithm excluded 21 immune features by applying the signaling-based penalization (table S3). These immune features were either (i) nonspecific signaling responses to ex vivo stimulation with LPS in cell subsets for which TLR4 expression is supported by little evidence [11 features, including the STAT1/3/5 signaling responses in T cell subsets and the inhibitor of nuclear factor κB (IκB) response to LPS stimulation in pDC andCD^4+^Tcells] or (ii) noncanonical signaling responses to IL-2/IL-6 or IFN-α stimulation [10 features, including extracellular signal–regulated kinase 1/2 (ERK1/2), riboprotein S6 (rpS6) and NF-κBphosphorylation in response to IFN-α stimulation, and adenosine 3′,5′-monophosphate response element–binding protein (CREB), rpS6, andMAPK-activated protein kinase 2 (MAPKPAK2) phosphorylation in response to IL-2/IL-6 stimulation].

### csEN components are proxies that reveal broader cellular programs of pregnancy-induced immune adaptations

The csEN algorithm provided a statistically robust predictive model by attributing positive or negative coefficients to a set of nonredundant immune features.An important step to further corroborate the csENmodel was the examination of its biological plausibility. Piecewise regression analysis of csEN outputs containing immune features associated with the highest coefficients revealed 25 particularly robust immune features that were further explored (Fig. 3, C and D, and fig. S2).

**Fig. 4:**
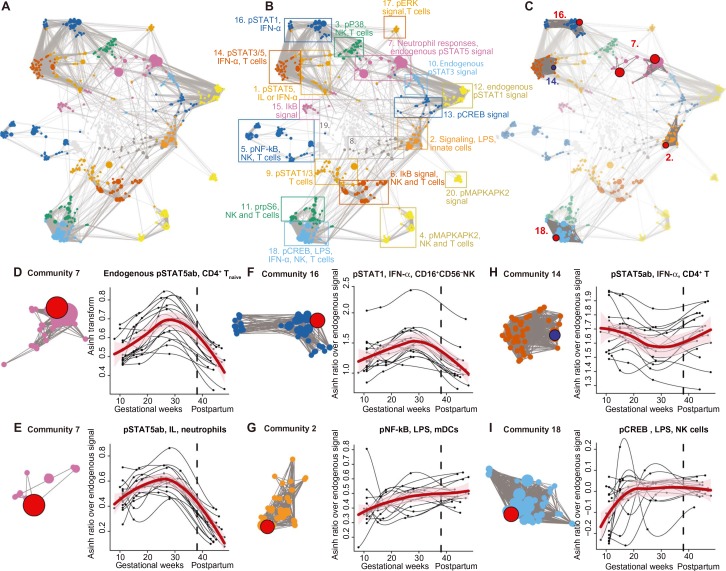
**csEN components reveal precisely timed cellular programs that characterize the dynamic changes of the peripheral immune system over the course of pregnancy.** (**A**) The correlation network segregated into 20 communities containing correlated immune features that changed in synchronicity during pregnancy. (**B**) The 20 communities were annotated on the basis of immune feature attributes (cell subset, stimulation, or signaling property) most commonly represented within each community. (**C**) Communities containing csEN components most predictive of gestational age are highlighted (red dots) and shown in table S3. Community numbers are indicated in red. (**D** to **I**) The five communities containing the most informative EN components of the csEN model (see fig. S2 and table S4). Communities are represented on the left. Graphs on the right depict csEN components (black lines represent each patient; red lines and red shadow represent median and 95% confidence interval, *n* =18).

An unsupervised clustering algorithm (*k*-means) segmented the correlation network into 20 distinct communities (Fig. 4A and the SupplementaryMaterials). Communities were described on the basis of the cell types, stimulation condition, or functional attributes that appearedmost frequently within each community (Fig. 4B). csEN components were nested in different communities and pointed to closely interrelated immune features behaving in synchrony over the course of pregnancy (Fig. 4C). Hence, csEN components can be viewed as statistically stringent proxies that can provide further insight into pregnancy-related immunological adaptations upon examination of their respective communities. We examined the five communities (16, 2, 18, 14, and 7, highlighted in Fig. 4C) that contained the most informative model components, as visualized by the largest (increasing strength of the correlation with the gestational age variable) and darkest (increasing absolute value of the csEN model coefficient) nodes on the graphical representation of the csEN model (fig. S2 and table S4).

Community 7 contained the two most informative components of the csENmodel, “endogenous pSTAT5ab signal in naïve CD4^+^ T cells” and “pSTAT5ab response to IL in neutrophils,” which were examined separately. Immune features most strongly correlated to the csEN component “endogenous pSTAT5ab signal in naïve CD4^+^ T cells” primarily pointed to endogenous pSTAT5ab signaling activities in T cell subsets. This community reflected important physiological cellular adaptations of the JAK/STAT5ab signaling pathway over the course of pregnancy. Compared with the postpartum state, endogenous STAT5ab signaling progressively increased over the course of pregnancy across several T cell subsets including memory CD4^+^, naive CD8^+^, memory CD8^+^, T cell receptor (TCR) γδ^+^ T, andCD25^+^ Forkhead box P3 (FoxP3)– (CD25^+^FoxP3^+^ T_regs_; Fig. 4D and fig. S3A). In contrast, the pSTAT5ab signal was not increased in innate immune cells during pregnancy (fig. S3). Community 7 also anchored in the csEN component “pSTAT5ab response to IL in neutrophils,”which was strongly correlated to immune features pointing at the expansion of neutrophils and their global hypersensitization to multiple immunological challenges during pregnancy including stimulation with IL-2 and IL-6, LPS, and IFN-α (Fig. 4E and fig. S3B).

The next four communities that were highly informative to the csEN model provided additional functional readouts for the capacity of specific immune cell subsets to respond to ex vivo stimulations during pregnancy. Community 16, anchored in the csEN component “pSTAT1 response to IFN-α in CD16^+^CD56−NK cells,” was primarily populated by immune features related to STAT1 tyrosine phosphorylation in response to IFN-α, an essential pathway for type-I IFN signal transduction (*23*). Compared with the postpartumstate, STAT1 signaling response to IFN-α increased in CD16^+^CD56−NK cells over the course of pregnancy (Fig. 4F). On further inspection, the analysis showed that multiple other immune cell subsets in community 16 displayed a coordinate STAT1 signaling response to IFN-α stimulation, including classical monocyte cells, myeloid DCs (mDCs), and CD8^+^ T cells (fig. S3C).

Community 2, anchored in the csEN component “pNF-κB response to LPS in mDCs,” was populated with immune features that primarily reflect signaling responses along the TLR4 pathway in myeloid cells upon stimulation with LPS (Fig. 4G). More specifically, activation (phosphorylation) of P38, MAPKAPK2, ERK1/2, rpS6, CREB, and NF-_κ_B by LPS was dampened in mDCs early in pregnancy compared with the postpartum state. Consistent with these findings, the IκB signal measured in LPS-stimulated mDCs decreased during pregnancy (fig. S3D). These results are suggestive of a coordinated dampening of TLR4 responses in mDCs in early pregnancy. These observations contrastwith the increased TLR4 response in neutrophils during pregnancy (Fig. 4E) and suggest that the modulation of the TLR4 pathway during gestation is time-dependent and cell type–specific.

Community 14 contained immune features that are correlated with the csEN model component “pSTAT5ab response to IFN-α in CD4^+^ T cells,” which decreased during pregnancy (Fig. 4H). The diminished capacity ofCD4^+^Tcells to mount a STAT5ab response to IFN-α reflected the increased endogenous STAT5ab signaling observed for this cell type (community 7, fig. S3E). Indeed, when controlled for changes in basal STAT5ab signaling, the pSTAT5ab signal measured in IFN-α-stimulated samples did not change during pregnancy (fig. S3E, black rectangle).This finding highlights the effect of basal signaling tone on the assessment of immune cell responses to in vitro stimulations, which are quantified as the difference in signaling activity between the stimulated and unstimulated (basal) conditions. A similar effect was observed for features within community 18, which correlated with the csEN component “pCREB response to LPS inNK cells” (Fig. 4I). In this case, a higher basal pCREB signal in NK cells primarily accounted for the decreased pCREB response to LPS observed early in pregnancy (fig. S3F, black rectangles).

### Intracellular pSTAT5ab signal provides an integrated readout of T cell responses to the systemic environment

Endogenous STAT5ab signaling responses in community 7 were particularly interesting because they were anchored on the most informative csEN model component and reflected in vivo functional behaviors of immune cell subsets throughout the course of pregnancy. These signaling responses were shared acrossmultipleTcell subsets,which suggest that common upstream mechanisms may regulate the STAT5abdependent activity and differentiation of these cell types during pregnancy. More than 20 cytokines and hormonal factors are known to activate the transcription factors STAT5a and STAT5b (referred together as STAT5ab) through cell type–specific combinations of receptor expression. STAT5ab was first described for its activation by prolactin (PRL) (*24*), a lactogenic hormone that increases during pregnancy. Among its many functions, PRL influences the proliferation and differentiation of B cells and T cells (*25*). This raises the question of whether the shared STAT5ab signaling observed inTcell subsets primarily reflects pregnancydependent changes in plasma PRL levels or, alternatively, represents an integrated cellular readout of multiple hormonal or immunological factors regulated by pregnancy.

**Fig. 5:**
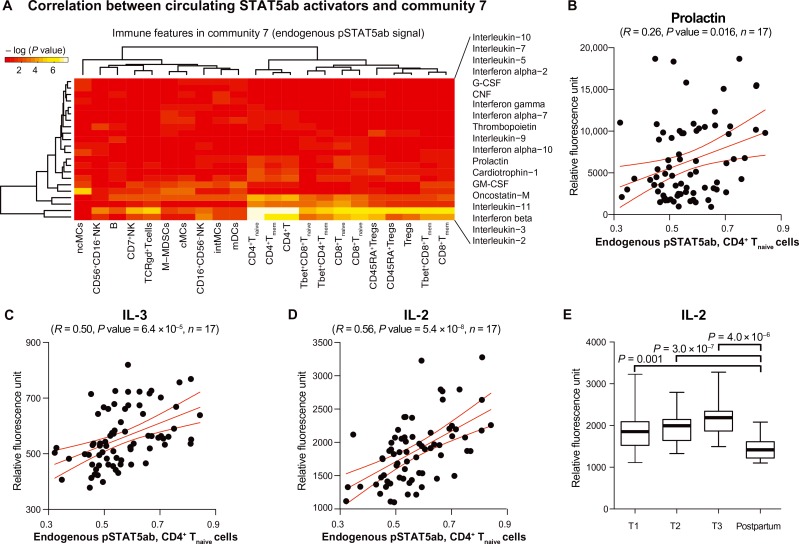
**Correlation between endogenous STAT5ab signaling and circulating plasmafactors.** (**A**) Heat map depicting the correlation between the plasma concentrations (relative fluorescence unit) of known activators of STAT5ab and immune features contained in community 7 (endogenous pSTAT5ab signaling in innate and adaptive cell subsets). Scale proportional to Spearman’s correlation *P* values (yellow indicates lower *P* values). (**B** to **D**) Correlations between the EN component “endogenous pSTAT5ab in naïve CD4^+^ T cells” and PRL (B), IL-3 (C), and IL-2 (D) plasma concentrations. The strongest correlation was observed between IL-2 and endogenous pSTAT5ab in naïve CD4^+^ T cells (*R* = 0.56, *P* = 5.4 × 10^−8^, *n* = 17). (**E**) Box plot depicting IL-2 plasma concentrations at each trimester (T1, T2, and T3) compared with their levels for the postpartum samples. IL-2 concentration increased during pregnancy andwas significantly higher at T1, T2, and T3 comparedwith the postpartumtime points (T1, *P* = 0.001; T2, *P*=3.0×10^−7^; T3, P = 4.0 × 10^−6^, unpaired t test). Median, interquartile range, and 5th to 95th percentiles are shown.

To address this question, we quantified the plasma levels of 18 circulating factors capable of activating the JAK/STAT5ab signaling pathway in peripheral leukocytes at each time point during pregnancy. The heatmap in Fig. 5A depicts the correlation between these 18 factors and endogenous STAT5ab activities captured in community 7. The data visually emphasize stronger associations between JAK/STAT5ab activators and the pSTAT5ab signal in T cell subsets rather than innate or B cells and indicate that multiple factors likely contribute to the shared STAT5ab response in T cell subsets. Although endogenous STAT5ab signaling in T cell subsets moderately correlated with PRL levels (*R* = 0.26, *P* = 0.016; Fig. 5B), correlations were stronger with immune mediators such as IL-3 (Fig. 5C) and IL-2 (Fig. 5D). The strongest correlation was observed between STAT5ab signaling in naïveCD4^+^Tcells and IL-2 (*R* = 0.56, *P* = 5.4 × 10^−6^), a cytokine that is mainly produced by activated T cells (*26*). Consistent with these findings, plasma IL-2 levels increased during pregnancy and were elevated at all trimesters compared with the postpartum levels (Fig. 5E). Together with findings from the csEN analysis, these results suggest a critical role for IL-2– dependent STAT5ab signaling pathways in modulating T cell function during pregnancy.

## DISCUSSION

This study applied high-dimensional mass cytometry to characterize with single-cell resolution the dynamic changes occurring in thematernal peripheral immune system over the course of a term pregnancy. We introduced a stringent analytical approach that takes advantage of the interconnected nature of the single-cell immunological data set to infer, as well as independently validate, a robust statistical model predicting gestational age during pregnancy. The model provided a set of biologically compelling immune features that captured the progression of the maternal immune system during pregnancy. Hence, the system-wide analysis of immune cell behaviors in peripheral blood revealed an immune clock of termpregnancy, a critical premise to detect chronological deviations associated with pathological pregnancies, such as preterm birth, in an accessible tissue compartment.

Analysis of peripheral blood samples with mass cytometry produced a highly correlated data set reflecting the modular biology of the immune system in which multiple cell subsets act in concert (*27*). The resulting high-content and interrelated data sets constitute an analytical challenge. A modified and extended version of the EN algorithm was implemented for the current analysis, which significantly increased the predictive power of the derived and validated csEN model (Fig. 3). The csEN algorithm allowed for an objective weighting of the observed immunological data based on the previous knowledge of signaling responses to receptor-specific ligands. In our data set, the csEN algorithm outperformed other widely used predictive methods. Integrating known biology into an ENanalysis is a powerful statistical approach suitable for the analysis of highly modular data sets.

The high-content mass cytometry data set segregated into communities of correlated immune features that behaved in synchrony over the course of pregnancy. The csENmodel components nested in such communities are statistically stringent immunological proxies that can reveal the broader biological characteristics of their respective communities. For example, several csENmodel components pointed at specific signaling responses that were shared across multiple cell lineages, including the increased endogenous STAT5ab activity among multiple T cell subsets or the increased STAT1 signaling response to IFN-α observed in a number of innate and adaptive immune cells. Alternatively, other csEN components pointed at coordinated signaling events within a specific immune cell subset (e.g., TLR4 signaling responses in mDCs). Hence, the analysis revealed entire cellular programs, rather than isolated mechanisms, that characterized the chronological progression of immune adaptations over the entire course of pregnancy.

The csEN model identified several features that are in agreement with previous work (*5*–*8*). With respect to innate cell function, our data indicate an overall enhancement of innate immune responses during pregnancy. Consistent with previous studies quantifying neutrophil abundance and proinflammatory functions during pregnancy (*6*), our findings suggest that the number of circulating neutrophils increased during pregnancy and that this abundance was correlated at the single-cell level with the progressive enhancement of the responsiveness of neutrophils to a variety of stimuli (including LPS, IFN-α, and IL-2/IL-6; Fig. 4E).

Similarly, within theNKcell compartment,we observed progressively enhanced STAT1 signaling responses to stimulation with IFN-α, a key mediator of defensive immunity against viral pathogens (Fig. 4F). A gestational age–dependent increase in STAT1 response to IFN-α also applied to several immune cell subsets including monocytes andmDCs. This result is consistent with previous in vitro and in vivo studies, which demonstrated that NK cell and monocyte responses to viral challenges are enhanced during pregnancy (*8*, *11*). Our data also suggested that basal CREB signaling in peripheral NK cells (both CD56^+^CD16− and CD56−CD16^+^ NK cell subsets) was increased early in pregnancy (Fig. 4I and fig. S3F). CD56^+^CD16− NK cells are the most abundant immune cells in the decidua during the first trimester of pregnancy, where they are critical for the normal development of the placenta and the establishment of feto-maternal tolerance (*28*, *29*). Several hypotheses regarding the origin of decidual NK cells have been considered, including the recruitment of mature peripheral CD56^+^CD16− NKcells, the differentiation of peripheral CD16^+^ CD56− NK cells, and the differentiation of progenitor cells within the decidua (*30*). Given the pivotal role for the transcription factor CREB in immune cell survival, it is possible that the increased basal CREB signaling tone observed early in pregnancy confers a survival advantage on peripheral NK cells, allowing them to contribute, asmature or precursor cells, to the pool of decidualNK cells.

In contrast to the STAT1 response to IFN-α observed in NK cells andmDCs, TLR4 signaling responses to LPS inmDCs were dampened early in pregnancy (Fig. 4G). TLR signaling can result in either proinflammatory or immunogenic versus tolerogenic programs, depending on the biologic context (*31*). Although the function ofDCs in maintaining tolerance toward the fetus has been studied extensively at the fetomaternal interface (*1*, *4*), fewer studies have focused on peripheral mDCs. Human peripheral mDCs express higher levels of tolerogenic surface proteins (including PD-L1, B7-H4, CD200, and CD200R) during the first trimester of pregnancy compared with the third trimester (*32*). Whether observed TLR4 hyporesponsiveness of mDCs contributes to peripheral tolerance toward fetal antigens early in pregnancy requires further investigation.

Some of our findings differ from previous descriptions of immune cell function during pregnancy. For example, Kraus *et al*. (*6*) reported broadly diminished cytokine expression in naïve CD4^+^ T cells and NK cells during pregnancy, whereas our data support an increased responsiveness of these cell types to certain extracellular ligands (e.g., STAT1 signaling responses to IFN-α). These discrepancies most likely result from technical differences in the assays used to examine immune cell function. In this regard, we opted for functional assays that emphasized short incubation times and minimal experimental manipulation of immune cells, whose functions were interrogated in the context of whole-blood samples.

Our analysis also enabled intriguing discoveries, especially with regard to endogenous T cell function. Pregnancy induced a robust and progressive increase in endogenous STAT5ab signaling across multiple T cell subsets, including CD25^+^FoxP3^+^ T_regs_, naïve and memory CD4^+^ and CD8^+^ T cells, and γδ T cells. In ovarian cells, STAT5ab plays a critical role downstream of PRL in maintaining progesterone levels during pregnancy (*33*). However, the importance of the progressively increased endogenous STAT5ab signaling activity in peripheral T cell subsets is unknown. Our data suggest that IL-2, perhaps in conjunction with other immune or hormonal mediators including IL-3 and PRL, is a prime candidate as an upstream activator of STAT5ab signaling in T cell subsets. Alternatively, observed changes in circulating IL-2 levels during pregnancy may be a downstream effect of antigen-dependent activation of CD4^+^ T cells, because activated CD4^+^ T cells are themain cellular sources of IL-2 (*26*, *34*). The IL-2/STAT5ab signaling pathway regulatesmany aspects of Tcell differentiation. IL-2–dependent STAT5ab activity is essential for the development of CD25^+^FoxP3^+^ T_regs_, which are critically implicated in the maintenance of feto-maternal tolerance during pregnancy (*35*). In pregnant mice, T_reg_ depletion via an IL-2 receptor (CD25)–specific antibody results in early resorption of the allogeneic fetus (*36*). Our finding of increased circulating IL-2 during pregnancy indicates that activated T cells may play an autocrine and/or paracrine role in enhancing immune regulation by T_regs_ during pregnancy. Thus, the progressive increase in endogenous STAT5ab signaling activitymay reflect at least in part an increasing requirement for the presence of T_regs_ throughout human pregnancy.

This study has several limitations. First, the cohort included women who all delivered at term but had various medical and obstetric histories. These clinical characteristics may of course modulate aspects of the immune response to pregnancy and contribute to observed interindividual variability. Our study was therefore designed to ask, “is there an immunological progression associated with gestational age in pregnancies that reach full term?” Despite some heterogeneity in the patient cohort, we were able to capture the chronology of immune adaptations over the course of term pregnancies and validate these findings in an independent cohort. On the other hand, the recruitment of participants from a single-care center limited the diversity of the study cohort. In the future, it will be important to test the generalizability of our approach and examine variations in the csENmodel associated with ethnic, racial, and socioeconomic differences. Second, the introduction of a signalingbased penalization favored ligand-specific signaling pathways by a 5:1 margin over other signaling responses. The rationale for introducing a signaling-based penalization was to reduce the impact of biologically less plausible immune features (such as induction of STAT3 by LPS in cell subsets not known to transduce TLR4 receptor signaling). This approach significantly improved the predictive power of the csEN model. However, a caveat inherent to cell signaling–based weighting is a decreased sensitivity for detecting biologically relevant but not particularly pronounced signaling responses. Last, although mass cytometry currently enables the simultaneous detection of more than 50 parameters, the technology remains limited to the analysis of preselected surface markers, intracellular responses and stimulation conditions. Future studies will expand on this effort and examine whether deeper phenotyping of specific cell subsets and interrogation of a greater range of signaling responseswill improve the predictive value of the csEN model.

Our study revealed a precisely timed chronology of immune adaptations in peripheral blood over the course of a term pregnancy. This finding was enabled by high-content, single-cell mass cytometry coupled with a csEN algorithm accounting for the modular structure of the immune system and previous knowledge. The study provides the conceptual backbone and the analytical framework to examine whether disruption of this chronology is a diagnostically useful characteristic of pretermbirth and other pregnancy-related pathologies.

## MATERIALS AND METHODS

### Study design

The aimof this observational study was to determine whether a precise chronology of pregnancy-related immunological adaptations is detectable from the mass cytometry analysis of peripheral immune cell phenotype and functional changes during pregnancy. The study was conducted at the Lucile Packard Children’s Hospital (Stanford, CA). The study was approved by the Institutional Review Board, and all participants signed an informed consent. Pregnant women receiving routine antepartum care were eligible for the study if they were 18 years of age and in their first trimester of pregnancy. Participants were followed longitudinally until 6 weeks after delivery of the fetus. Comorbidities included hypertension (3), asthma (2), hypothyroidism (2), and autoimmune hepatitis (1).Only participants that delivered at term(≥37 weeks of gestation) were included in the analysis. Pregnancy complications included mild gestational hypertension (2) and preeclampsia (2). Demographics and pregnancy characteristics for the 28 participants included in the analysis are summarized in Table 1.

### Derivation of mass cytometry immune features

Whole-blood samples collected at three time points during pregnancy (an early-, a mid-, and a late-pregnancy time point) and 6 weeks postpartum were stimulated for 15min with either LPS, IFN-α, or a cocktail containing IL-2 and IL-6, or left unstimulated. Samples were then processed using a standardized protocol for fixation (Smart Tube Inc.), barcoding, and antibody staining of whole-blood samples for mass cytometry analysis (see the Supplementary Materials) (*13*). Mass cytometry data from each sample were manually gated into 24 immune cell types of interest (fig. S1). Three categories of immune features were derived:

Cell frequency features: Cell frequencieswere expressed as a percentage of gated singlets in the case of neutrophils and as a percentage of mononuclear cells (CD45^+^CD66^−^) in the case of all other cell types.

Endogenous signaling immune features: Endogenous intracellular signaling activitieswere derived from the analysis of unstimulated blood samples. The signal intensity of the following functional markers was simultaneously quantified per single cell: phospho (p) STAT1, pSTAT3, pSTAT5, pNFkB, total IkB, pMAPKAPK2, pP38, prpS6, pERK1/2, and pCREB. For each cell type, signaling immune featureswere calculated as the median signal intensity (arcsinh transformed value) of each signaling protein.

Intracellular signaling response features: The signal intensity of all functional markers was analyzed from samples stimulated with LPS, IFN-α, or IL. For each cell type, signaling responses were calculated as the difference in median signal intensity (arcsinh transformed value) of each signaling protein between the stimulated and unstimulated conditions.

### Proteomic analysis of circulating plasma factors

A total of 1310 proteins were assayed from plasma samples collected at each time point using a highly multiplexed, aptamer-based platform (SomaLogic Inc.) (*37*). See details in the Supplementary Materials.

### Statistical analysis

#### Multivariate modeling of mass cytometry data using a cell signaling–based matrix

The csEN algorithm was adapted from the existing EN algorithm (*18*) to allow accounting for the influence of previous knowledge of intracellular signal transduction on the generation of the mass cytometry data set. For a matrix *X* of all immune features and a vector of estimated gestational age at time of sampling *Y*, a multivariate model was developed to calculate the coefficients β for each entity in *X* to minimize the overall differences from *Y*: *L*(β) = |*Y* – *X* β |^2^. An *L*_1_ regularization (*38*) was applied on the β coefficients to reduce the model complexity, such that *L*(β) = |*Y* − *X*β |^2^ + λ_1_|β |_1_, where λ_1_ is selected by cross-validation. This produces a sparse model in which only a limited number of features are used. However, this approach is not ideal for the analysis of the highly interrelated mass cytometry data set, because it would select only representatives of communities of correlated featureswhile disregarding highly correlated but potentially biologically relevant features. This limitation is addressed by using an additional *L*_2_ regularization (*18*) to allow the inclusion of highly correlated measurements: *L*(β) = |*Y* − *X*β|^2^ + λ_1_|β|_1_ + λ_2_|β|_2_, where λ_1_ and λ_2_ are selected by cross-validation.

To account for the fact that previous biological knowledge of intracellular signal transduction influences the selection ofmarkers included in themass cytometry panel, we introduced a previously unknown term into the model that allowed prioritizing receptor-specific signaling responses selected a priori by a cell signaling–based matrix (described in Fig. 2 and table S2):

$$L(\text{β})={\left|Y-k\psi X\text{β}\right|}^{2}+\lambda_{1}{\left|\text{β}\right|}_{1}+\lambda_{2}{\left|\text{β}\right|}_{2}$$

$$\text{where}\;k=\left\{ \begin{array}{l} \text{1 if feature is selected}\\ \varphi \;\text{if feature is}\;\text{not selected} \end{array}\right.$$

The four freeparametersof themodel (λ_1_, λ_2_,φ, and ψ)were optimized using a gradient-free optimization algorithm (*39*). This optimization algorithm maximized a score function derived from the results of the cross-validation analysis evaluating the correlation between the model and the gestational age at time of sampling (see the csEN algorithm evaluation section in the Supplementary Materials). The optimization algorithmwas initialized using an exhaustive search with a uniform grid for all values in [0, 1]. Optimization results for φ and ψ were φ ~ 0.21 and ψ ~ 1.91, indicating that a ~5:1 prioritization of knowledge-based signaling responses provided the most robust model. Algorithm evaluation, model reduction, handling of missing values, post hoc analysis, and visualization of the immune network are described in the Supplementary Materials.

### Reproducibility and data availability

Raw data, processed data, and source code for reproduction of the results are publicly available at http://flowrepository.org/id/FR-FCMZY3Q and http://flowrepository.org/id/FR-FCM-ZY3R for the original and validation studies, respectively.

## SUPPLEMENTARY MATERIALS

immunology.sciencemag.org/cgi/content/full/2/15/eaan2946/DC1

Materials and Methods

Fig. S1. Gating strategy of immune cell subsets.

Fig. S2. Reduced csEN model components.

Fig. S3. Time-dependent changes in csEN model components are reflected across communities of interrelated immune features.

Table S1. Antibody panel used for mass cytometry analysis.

Table S2. Signaling responses prioritized in the signaling-based penalization matrix by a 5:1 margin.

Table S3. Features excluded from the csEN model as compared with the non–signaling-based EN model.

Table S4. Reduced csEN model components.

References (*40*–*49*)

## Supplementary Material

An immune clock of human pregnancy
