# Supplementary material for: An immune clock of human pregnancy
Source: Sci Immunol. 2017 Sep 1;2(15):eaan2946. doi: 10.1126/sciimmunol.aan2946 (PMC5701281; doi:10.1126/sciimmunol.aan2946)
Supplement: An immune clock of human pregnancy [file STM-02-eaan2946-s001.pdf]

immunology.sciencemag.org/cgi/content/full/2/15/eaan2946/DC1

Science Immunology

Supplementary Materials for

An immune clock of human pregnancy

Nima Aghaeepour, Edward A. Ganio, David Mcilwain, Amy S. Tsai, Martha Tingle, Sofie Van Gassen, Dyani K. Gaudilliere, Quentin Baca, Leslie McNeil, Robin Okada, Mohammad S. Ghaemi, David Furman, Ronald J. Wong, Virginia D. Winn, Maurice L. Druzin, Yaser Y. El-Sayed, Cecele Quaintance, Ronald Gibbs, Gary L. Darmstadt, Gary M. Shaw, David K. Stevenson, Robert Tibshirani, Garry P. Nolan, David B. Lewis, Martin S. Angst, Brice Gaudilliere*

*Corresponding author. Email: gbrice@stanford.edu

Published 1 September 2017, *Sci. Immunol.* **2**, eaan2946 (2017)
DOI: 10.1126/sciimmunol.aan2946

This PDF file includes:

Materials and Methods
Fig. S1. Gating strategy of immune cell subsets.
Fig. S2. Reduced csEN model components.
Fig. S3. Time-dependent changes in csEN model components are reflected across communities of interrelated immune features.
Table S1. Antibody panel used for mass cytometry analysis.
Table S2. Signaling responses prioritized in the signaling-based penalization matrix by a 5:1 margin.
Table S3. Features excluded from the csEN model as compared with the non–signaling-based EN model.
Table S4. Reduced csEN model components.
References (*40*–*49*)

SUPPLEMENTARY MATERIALS

Materials and Methods

Mass cytometry

Sample barcoding

To minimize the effect of experimental variability on mass cytometry measurements between samples from different time points, samples corresponding to an entire time series were barcoded, stained, and run simultaneously on the mass cytometry instrument (40). Because the csEN model was built against the gestational age at time of sampling, this barcoding strategy minimized the impact of experimental variability on the csEN model's false-positive rate.

Antibodies

The mass cytometry antibody panel contained 23 antibodies that were used for cell phenotyping and 10 antibodies that were used for functional analysis of signaling responses (Table S1). Antibodies were either obtained pre-conjugated from the manufacturer (Fluidigm) or were conjugated in-house with the appropriate metal isotopes. Purified unconjugated antibodies in protein-free PBS carrier were labeled using the MaxPAR antibody conjugation kit (Fluidigm) according to the manufacturer's instructions. All antibodies used in the analysis (conjugated in-house as well as those obtained pre-conjugated) were titrated and validated on samples that were processed identically to the samples used in the study. Antibodies were used at concentrations listed in Table S1.

Supplementary statistical analysis and data visualization

csEN Algorithm evaluation

A cross-validation procedure was applied to test the generalizability of the multivariate models to previously unseen samples. To account for interdependencies between samples from the same patient, for each cross-validation iteration all samples corresponding to the entire time series from one patient were excluded from the cohort used to build the model. The resulting model was used for estimating the gestational age at the time of sampling for the excluded patient. The procedure was repeated until an estimation of the gestational age was obtained for time points of all patients. This cross-validation procedure was applied for testing a range of

algorithms representing common alternative learning algorithms, including randomForest (19), k-Nearest Neighbors (20), Support Vector Machines (21), and LASSO (22). These algorithms were used with the default parameterizations documented for each software package.

Model reduction

A bootstrapping procedure was used to identify the most stringent components of the EN model that were examined for further biological interpretation. One hundred bootstrap iterations were performed during which a subset of the patients equal to the size of the full dataset was selected randomly with replacement. All time points of each selected patient were included in the model for cross-validation. Piece-wise regression (41) between the number of features (calculated by applying a range of thresholds to the mean coefficient of each measurement across all bootstrap iterations) and the final results of the model was used to select the number of features.

Handling of missing values and post-hoc analysis

Samples collected from two patients provided insufficient volume to allow for stimulation with extracellular ligands. Values for the intracellular signaling response features for these two patients were set to the average of the respective values from the entire cohort. Plasma samples from one patient were excluded from the proteomic analysis for technical reasons.

Given the potential effect of autoimmune hepatitis and preeclampsia on systemic immune responses measured during pregnancy, the analysis was repeated excluding patients with these medical conditions. Cross-validation of the csEN model remained highly significant when excluding samples from the patient with autoimmune hepatitis and preeclampsia from the training set ($R = 0.89$, $p = 2.2*10^{-16}$). Excluding samples from the patient with preeclampsia from the validation set improved the strength of the csEN model validation ($R = 0.68$, $p = 9.1*10^{-5}$).

Correlation network

Spearman correlation analysis was performed between all pairs of immune features. The correlation network consists of a graph on which each edge represents a significant correlation between the two respective immune features ($p < 0.05$ after Bonferroni adjustment, Fig. 2). The graph layout was calculated using the t-

SNE algorithm (42). To visualize the modularity of the network (*i.e.* to highlight groups of correlated immune features), communities of correlated immune features were calculated using the K-means algorithm applied to the entire correlation matrix.

Longitudinal Data Visualization

Longitudinal result from the full model (Fig. 2 E and G) and individual features (Fig. 4 D to I, Fig. S3) were visualized using a smoothing spline to each patient (gray) and the full dataset (red) (43). A 95% confidence interval is displayed for the full dataset.

Proteomic analysis of circulating plasma factors

The assay quantifies proteins over a wide dynamic range (> 8 log) and with high precision (median coefficient of variation <5%) using chemically modified aptamers with slow off-rate kinetics (SOMAmer reagents). Each SOMAmer reagent is a unique, high-affinity, single-strand DNA endowed with functional groups mimicking amino acid side chains. In brief, biological sample were incubated on a single 96-well plate with a mixture of 1,310 SOMAmer reagents. Two sequential bead-based immobilization and washing steps eliminated non-specifically bound proteins, unbound proteins, and unbound SOMAmer reagents from protein target-bound SOMAmer reagents. After eluting SOMAmer reagents from the target proteins, the fluorescently labeled SOMAmer reagents were quantified on an Agilent hybridization array (Agilent Technologies, Santa Clara, CA). Data were normalized according to assay data quality control (QC) procedures defined in the good laboratory practice (GLP) quality system of SomaLogic, Inc. Specifically, normalization controlled for “bulk” signal intensity biases either introduced by differential hybridization efficiency or differential sample dilution (among other collection protocol artifacts). Differential hybridization efficiency was captured by a set of control sequences introduced into the assay prior to hybridization. Adjustments for collection protocol artifacts were made by using the median of the ratio of median signal levels in each sample to the median signal level for the SOMAmer reagent across all samples as a scale factor. Typical normalization scale factors are close to unity and quality control (QC) acceptance criteria require scale factors for a sample to fall in the range of 0.4 to 2.5. Samples from one patient did not pass QC and were excluded from the analysis.

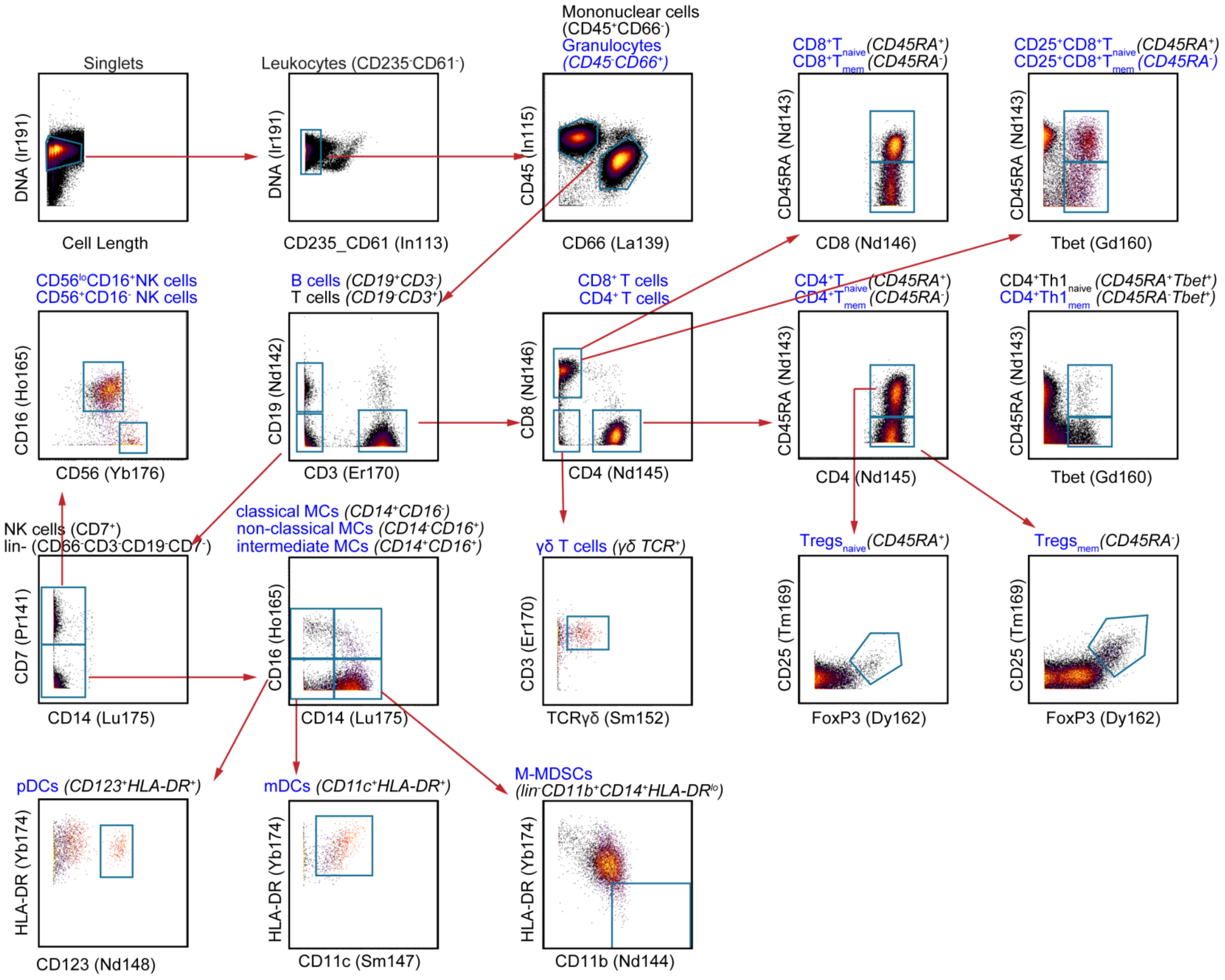

Fig. S1. Gating strategy of immune cell subsets. Two-dimensional flow cytometry plots are shown for a representative patient sample. Gating was performed using Cytobank software (www.cytobank.org). Twenty-four innate and adaptive cell subsets were manually gated and included in the analysis (blue font). Abbreviations are NK: Natural Killer, cMC: classical monocytes, ncMC: non-classical monocytes, intMC: intermediate monocytes, pDC: plasmacytoid dendritic cell, mDC: myeloid dendritic cell, mem: memory, T_{regs}: regulatory T cells, TCR: T cell receptor, and M-MDSC: monocytic myeloid derived suppressor cells.

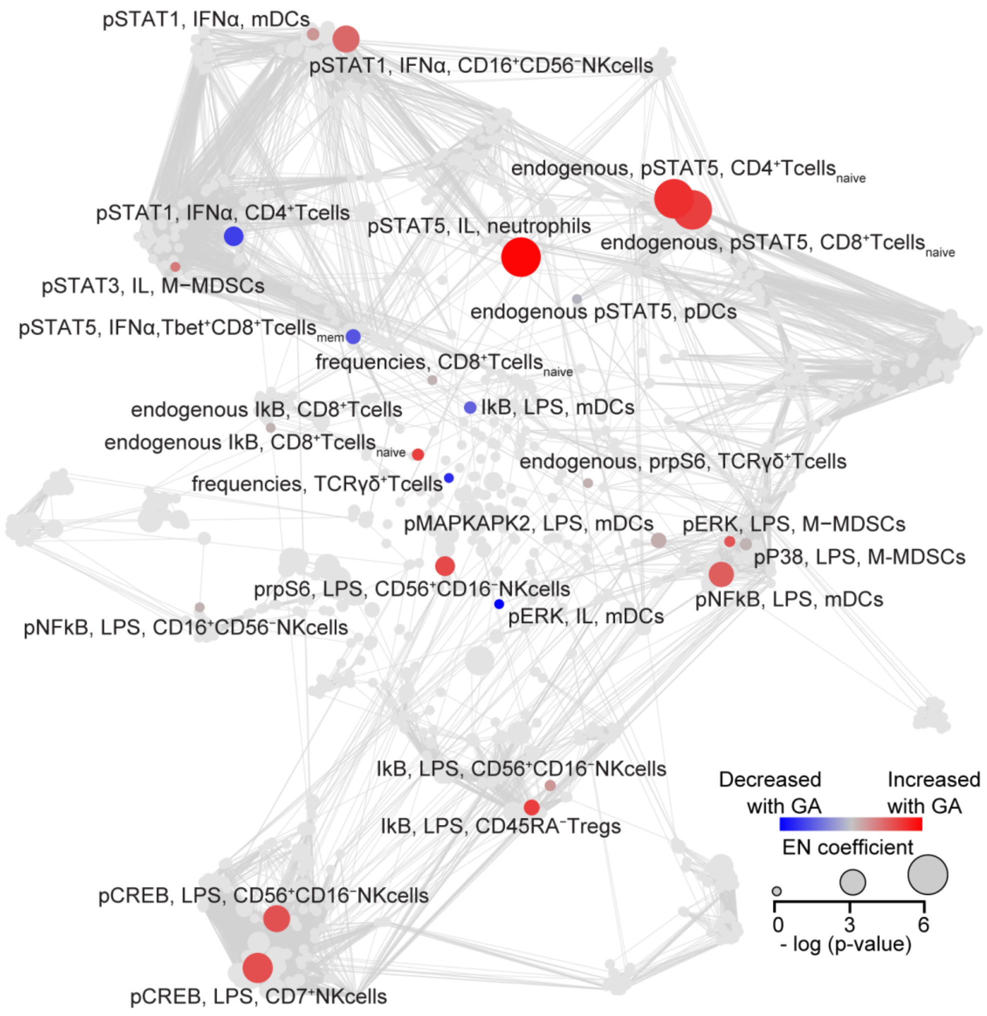

Fig. S2. Reduced csEN model components. Piece-wise regression analysis produced a reduced csEN model containing the most informative components of the csEN model predicting gestational age at time of sampling. csEN model components overlaid on the immune correlation network are colored in red (blue) if they trend upward (downward) during pregnancy. Color intensity is proportional to the absolute value of csEN coefficients. Dot size indicates the strength of the correlation between csEN components and the gestational age at time of sampling (Spearman's coefficient). Components of the csEN model were ranked by degree of importance (Table S4) according to the product of the csEN coefficient by the strength of the Spearman's correlation with getatsional age (-log(p-value)).

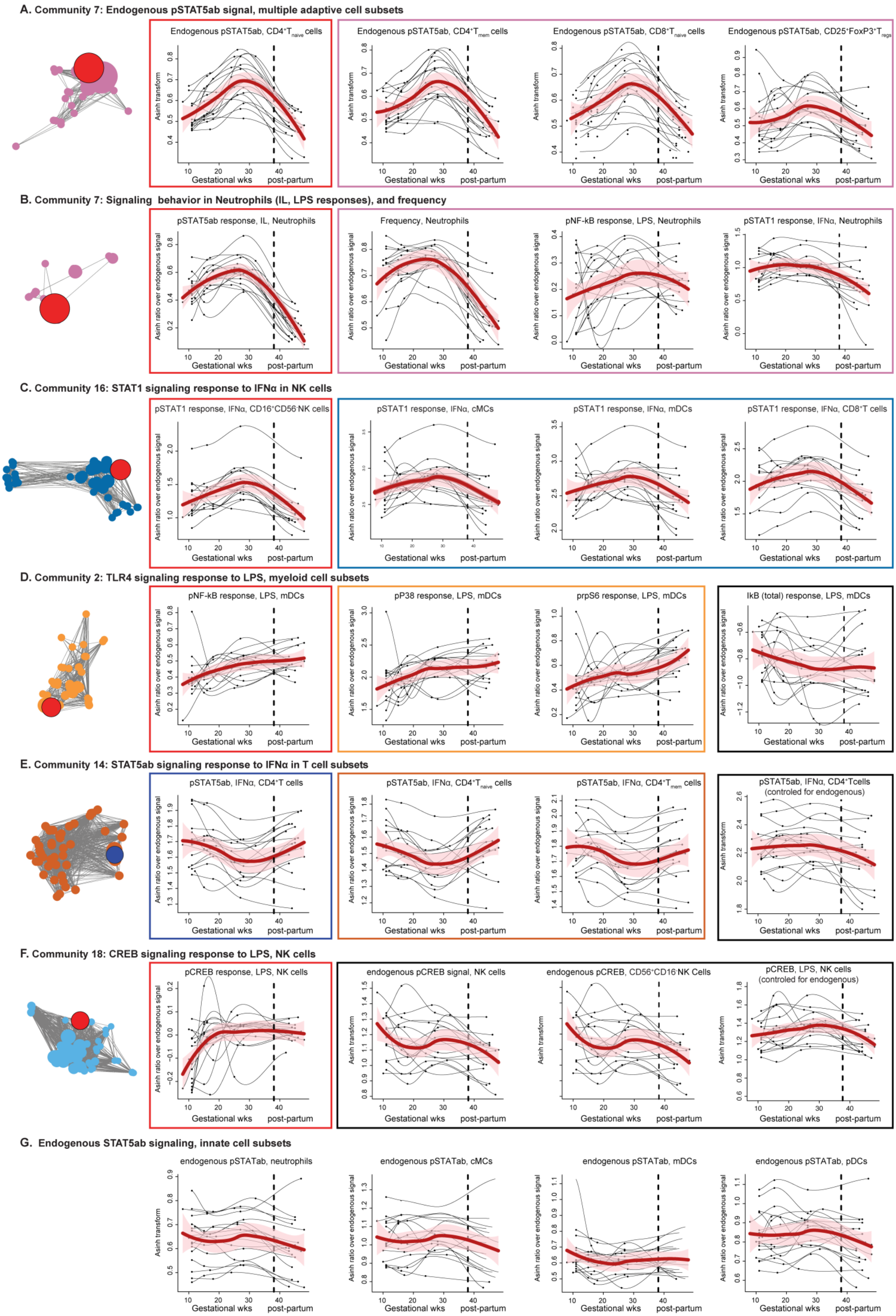
A. Community 7: Endogenous pSTAT5ab signal, multiple adaptive cell subsets
Endogenous pSTAT5ab, CD4+T naive cells
Endogenous pSTAT5ab, CD4+T mem cells
Endogenous pSTAT5ab, CD8+T naive cells
Endogenous pSTAT5ab, CD25+FoxP3+T regs
Asinh transform
Gestational wks
post-partum
B. Community 7: Signaling behavior in Neutrophils (IL, LPS responses), and frequency
pSTAT5ab response, IL, Neutrophils
Frequency, Neutrophils
pNF-kB response, LPS, Neutrophils
pSTAT1 response, IFNα, Neutrophils
Asinh ratio over endogenous signal
C. Community 16: STAT1 signaling response to IFNα in NK cells
pSTAT1 response, IFNα, CD16+CD56-NK cells
pSTAT1 response, IFNα, cMCs
pSTAT1 response, IFNα, mDCs
pSTAT1 response, IFNα, CD8+T cells
D. Community 2: TLR4 signaling response to LPS, myeloid cell subsets
pNF-kB response, LPS, mDCs
pP38 response, LPS, mDCs
prpS6 response, LPS, mDCs
IkB (total) response, LPS, mDCs
E. Community 14: STAT5ab signaling response to IFNα in T cell subsets
pSTAT5ab, IFNα, CD4+T cells
pSTAT5ab, IFNα, CD4+T naive cells
pSTAT5ab, IFNα, CD4+T mem cells
pSTAT5ab, IFNα, CD4+Tcells
(controled for endogenous)
F. Community 18: CREB signaling response to LPS, NK cells
pCREB response, LPS, NK cells
endogenous pCREB signal, NK cells
endogenous pCREB, CD56+CD16-NK Cells
pCREB, LPS, NK cells
(controled for endogenous)
G. Endogenous STAT5ab signaling, innate cell subsets
endogenous pSTATab, neutrophils
endogenous pSTATab, cMCs
endogenous pSTATab, mDCs
endogenous pSTATab, pDCs

Fig. S3. Time-dependent changes in csEN model components are reflected across communities of interrelated immune features. (A) Changes in the csEN component “endogenous pSTAT5ab signal in naïve $CD4^+$ T cells” (red rectangle) were reflected in several T cell subsets within community 7. **(B)** The csEN component “pSTAT5ab response to IL in neutrophils” was interrelated with the expansion of neutrophils over the course of pregnancy and increased neutrophil responses to LPS (pNF-κB signal) and IFNα (pSTAT1 signal). **(C)** The csEN component “pSTAT1 response to IFNα stimulation in $CD16^+CD56^-$NK cells” was reflected in several innate and adaptive immune cell subsets within community 16, including cMCs, mDCs and $CD8^+$T cells. **(D)** The csEN component “pNF-κB response to LPS in mDCs” became less dampened during pregnancy and correlated with multiple signaling elements of the TLR4 pathway within community 2. The decrease in total IκB signal in response to LPS measured simultaneously in mDCs is shown on the right (black rectangle). **(E)** The decrease in pSTAT5ab response to IFNα in multiple T cell subsets (community 14) primarily reflected the increase in endogenous pSTAT5ab signal observed for immune features in community 7. As such, when controlled for the endogenous pSTAT5ab signal, the pSTAT5ab signal in the IFNα stimulation condition did not change during pregnancy (black rectangle). **(F)** Community 18 contained two csEN components pointing at a decrease in pCREB signaling response to LPS in NK cell subsets early in pregnancy. The decreased pCREB response to LPS in these cell subsets primarily reflected an increase in basal (endogenous) pCREB signal early in pregnancy (black rectangle). When controlled for changes in basal pCREB signal, the pCREB signal in the LPS stimulation condition did not change during pregnancy (black rectangle). **G)** Little or no change was observed in endogenous pSTAT5ab signals in innate immune cells.

Table S1. Antibody panel used for mass cytometry analysis.

Antigen	Supplier	Symbol	Atomic Mass	Clone	Comment
**Barcode 1**		Pd	102		Barcode
**Barcode 2**		Pd	104		Barcode
**Barcode 3**		Pd	105		Barcode
**Barcode 4**		Pd	106		Barcode
**Barcode 5**		Pd	108		Barcode
**Barcode 6**		Pd	110		Barcode
**CD235ab**	Biolegend	In	113	HIR2	phenotype
**CD61**	BD	In	113	VI-PL2	phenotype
**CD45**	Biolegend	In	115	HI30	phenotype
**CD66**	BD	La	139	CD66α-B1.1	phenotype
**CD7**	BD	Pr	141	M-T701	phenotype
**CD19**	Fluidigm	Nd	142	HIB19	phenotype
**CD45RA**	Fluidigm	Nd	143	HI100	phenotype
**CD11b**	Fluidigm	Nd	144	ICRF44	phenotype
**CD4**	Fluidigm	Nd	145	RPA-T4	phenotype
**CD8a**	Fluidigm	Nd	146	RPA-T8	phenotype
**CD11c**	Fluidigm	Sm	147	Bu15	phenotype
**CD123**	Biolegend	Nd	148	6H6	phenotype
**pCREB**	CST	Sm	149	87G3	function
**pSTAT5**	Fluidigm	Nd	150	47	function
**pP38**	CST	Eu	151	36/p38/pT18	function
**TCRγδ**	Fluidigm	Sm	152	GL3	phenotype
**pSTAT1**	Fluidigm	Eu	153	58D6	function
**pSTAT3**	BD	Sm	154	4/P pY705	function
**prpS6**	CST	Gd	155	N7-548	function
**CD33**	Fluidigm	Gd	158	WM53	phenotype
**pMAPKAPK**	Fluidigm	Tb	159	27B7	function
**Tbet**	Fluidigm	Gd	160	4B10	phenotype
**FoxP3**	Fluidigm	Dy	162	PCH101	phenotype
**IκB**	Fluidigm	Dy	164	L35A5	function
**CD16**	Fluidigm	Ho	165	3G8	phenotype
**pNFκB**	Fluidigm	Er	166	K10-	function
**pERK1/2**	CST	Er	167	D13.14.4E	function
**CD25**	Fluidigm	Tm	169	2A3	phenotype
**CD3**	Fluidigm	Er	170	UCHT1	phenotype
**CD15**	Fluidigm	Yb	172	W6D3	phenotype
**HLA-DR**	Fluidigm	Yb	174	L243	phenotype
**CD14**	Fluidigm	Yb	175	M52E	phenotype
**CD56**	Fluidigm	Yb	176	NCAM16.2	phenotype
**DNA1**		Ir	191		
**DNA2**		Ir	192		

Table S2. Signaling responses prioritized in the signaling-based penalization matrix by a 5:1 margin.

A.

IFNα	pCREB	pERK1/2	IkB	pMK2	pNFkB	pP38	prpS6	pSTAT1	pSTAT3	pSTAT5
Granulocytes								✓	✓	✓
cMCs								✓	✓	✓
ncMCs								✓	✓	✓
intMCs								✓	✓	✓
M-MDSCs								✓	✓	✓
mDCs								✓	✓	✓
pDCs								✓	✓	✓
NKcells								✓	✓	✓
CD16$^+$CD56$^-$NKcells								✓	✓	✓
CD56$^+$CD16$^-$NKcells								✓	✓	✓
CD4$^+$Tcells								✓	✓	✓
CD4$^+$Tcells$_{mem}$								✓	✓	✓
CD4$^+$Tcells$_{naive}$								✓	✓	✓
Tbet$^+$CD4$^+$Tcells								✓	✓	✓
TCRγδ$^+$Tcells								✓	✓	✓
CD45RA$^-$Tregs								✓	✓	✓
CD45RA$^+$Tregs								✓	✓	✓
Tregs								✓	✓	✓
CD8$^+$Tcells								✓	✓	✓
CD8$^+$Tcells$_{mem}$								✓	✓	✓
CD8$^+$Tcells$_{naive}$								✓	✓	✓
Tbet$^+$CD8$^+$Tcells$_{mem}$								✓	✓	✓
Tbet$^+$CD8$^+$Tcells$_{naive}$								✓	✓	✓
Bcells								✓	✓	✓

C.

LPS	pCREB	pERK1/2	IkB	pMK2	pNFkB	pP38	prpS6	pSTAT1	pSTAT3	pSTAT5
Granulocytes	✓	✓	✓	✓	✓	✓	✓			
cMCs	✓	✓	✓	✓	✓	✓	✓			
ncMCs	✓	✓	✓	✓	✓	✓	✓			
intMCs	✓	✓	✓	✓	✓	✓	✓			
M-MDSCs	✓	✓	✓	✓	✓	✓	✓			
mDCs	✓	✓	✓	✓	✓	✓	✓			
pDCs										
NKcells	✓	✓	✓	✓	✓	✓	✓			
CD16$^+$CD56$^-$NKcells	✓	✓	✓	✓	✓	✓	✓			
CD56$^+$CD16$^-$NKcells	✓	✓	✓	✓	✓	✓	✓			
CD4$^+$Tcells										
CD4$^+$Tcells$_{mem}$										
CD4$^+$Tcells$_{naive}$										
Tbet$^+$CD4$^+$Tcells										
TCRγδ$^+$Tcells										
CD45RA$^-$Tregs	✓	✓	✓	✓	✓	✓	✓			
CD45RA$^+$Tregs	✓	✓	✓	✓	✓	✓	✓			
Tregs	✓	✓	✓	✓	✓	✓	✓			
CD8$^+$Tcells										
CD8$^+$Tcells$_{mem}$										
CD8$^+$Tcells$_{naive}$										
Tbet$^+$CD8$^+$Tcells$_{mem}$										
Tbet$^+$CD8$^+$Tcells$_{naive}$										
Bcells										

B.

IL (IL-2/IL-6)	pCREB	pERK1/2	IkB	pMK2	pNFkB	pP38	prpS6	pSTAT1	pSTAT3	pSTAT5
Granulocytes		✓						✓	✓	✓
cMCs		✓						✓	✓	✓
ncMCs		✓						✓	✓	✓
intMCs		✓						✓	✓	✓
M-MDSCs		✓						✓	✓	✓
mDCs		✓						✓	✓	✓
pDCs		✓						✓	✓	✓
NKcells		✓						✓	✓	✓
CD16$^+$CD56$^-$NKcells		✓						✓	✓	✓
CD56$^+$CD16$^-$NKcells		✓						✓	✓	✓
CD4$^+$Tcells		✓						✓	✓	✓
CD4$^+$Tcells$_{mem}$		✓						✓	✓	✓
CD4$^+$Tcells$_{naive}$		✓						✓	✓	✓
Tbet$^+$CD4$^+$Tcells		✓						✓	✓	✓
TCRγδ$^+$Tcells		✓						✓	✓	✓
CD45RA$^-$Tregs		✓						✓	✓	✓
CD45RA$^+$Tregs		✓						✓	✓	✓
Tregs		✓						✓	✓	✓
CD8$^+$Tcells		✓						✓	✓	✓
CD8$^+$Tcells$_{mem}$		✓						✓	✓	✓
CD8$^+$Tcells$_{naive}$		✓						✓	✓	✓
Tbet$^+$CD8$^+$Tcells$_{mem}$		✓						✓	✓	✓
Tbet$^+$CD8$^+$Tcells$_{naive}$		✓						✓	✓	✓
Bcells		✓						✓	✓	✓

D.

Endogenous	pCREB	pERK1/2	IkB	pMK2	pNFkB	pP38	prpS6	pSTAT1	pSTAT3	pSTAT5
Granulocytes	✓	✓	✓	✓	✓	✓	✓	✓	✓	✓
cMCs	✓	✓	✓	✓	✓	✓	✓	✓	✓	✓
ncMCs	✓	✓	✓	✓	✓	✓	✓	✓	✓	✓
intMCs	✓	✓	✓	✓	✓	✓	✓	✓	✓	✓
M-MDSCs	✓	✓	✓	✓	✓	✓	✓	✓	✓	✓
mDCs	✓	✓	✓	✓	✓	✓	✓	✓	✓	✓
pDCs	✓	✓	✓	✓	✓	✓	✓	✓	✓	✓
NKcells	✓	✓	✓	✓	✓	✓	✓	✓	✓	✓
CD16$^+$CD56$^-$NKcells	✓	✓	✓	✓	✓	✓	✓	✓	✓	✓
CD56$^+$CD16$^-$NKcells	✓	✓	✓	✓	✓	✓	✓	✓	✓	✓
CD4$^+$Tcells	✓	✓	✓	✓	✓	✓	✓	✓	✓	✓
CD4$^+$Tcells$_{mem}$	✓	✓	✓	✓	✓	✓	✓	✓	✓	✓
CD4$^+$Tcells$_{naive}$	✓	✓	✓	✓	✓	✓	✓	✓	✓	✓
Tbet$^+$CD4$^+$Tcells	✓	✓	✓	✓	✓	✓	✓	✓	✓	✓
TCRγδ$^+$Tcells	✓	✓	✓	✓	✓	✓	✓	✓	✓	✓
CD45RA$^-$Tregs	✓	✓	✓	✓	✓	✓	✓	✓	✓	✓
CD45RA$^+$Tregs	✓	✓	✓	✓	✓	✓	✓	✓	✓	✓
Tregs	✓	✓	✓	✓	✓	✓	✓	✓	✓	✓
CD8$^+$Tcells	✓	✓	✓	✓	✓	✓	✓	✓	✓	✓
CD8$^+$Tcells$_{mem}$	✓	✓	✓	✓	✓	✓	✓	✓	✓	✓
CD8$^+$Tcells$_{naive}$	✓	✓	✓	✓	✓	✓	✓	✓	✓	✓
Tbet$^+$CD8$^+$Tcells$_{mem}$	✓	✓	✓	✓	✓	✓	✓	✓	✓	✓
Tbet$^+$CD8$^+$Tcells$_{naive}$	✓	✓	✓	✓	✓	✓	✓	✓	✓	✓
Bcells	✓	✓	✓	✓	✓	✓	✓	✓	✓	✓

For each stimulation condition, receptor-specific signaling responses were emphasized based on review of the literature describing canonical intracellular signaling pathways activated downstream of IFNα, LPS, and IL-2/IL-6 (only recent reviews and seminal studies are included in the supporting references). Prioritization tables were constructed before the analysis. The matrix emphasized: **(A)** the phosphorylation of STAT1, STAT3 and STAT5 in all adaptive and innate immune cells in response to IFNα stimulation (23); **(B)** The phosphorylation of STAT1, STAT3, STAT5, and ERK1/2 MAPK in all adaptive and innate immune cells in response to the IL cocktail containing IL-2 and IL-6 (34,44); **(C)** the phosphorylation of P38 MAPK, MAPKAPK2, ERK1/2, rpS6, CREB, and NF-κB and total IκB signal in all innate immune cells (except pDCs), and in regulatory T cells in response to LPS stimulation condition (45-49) **(D)** All endogenous signaling responses were emphasized equally.

Table S3. Features excluded from the csEN model as compared with the non–signaling-based EN model.

Features excluded from csEN model
IkB response, LPS, $CD4^{+}Tcells_{naive}$
pSTAT1 response, LPS, $CD4^{+}Tcells_{naive}$
pSTAT5 response, LPS, $CD4^{+}Tcells_{naive}$
pSTAT1 response, LPS, $CD45RA^{+}$Tregs
pSTAT3 response, LPS, $CD45RA^{+}$Tregs
pSTAT5 response, LPS, $CD45RA^{+}$Tregs
pNFkB response, LPS, pDCs
pSTAT5 response, LPS, pDCs
pSTAT5 response, LPS, $Tbet^{+}CD4^{+}Tcells_{mem}$
pSTAT5 response, LPS, $Tbet^{+}CD8^{+}Tcells_{naive}$
pSTAT5 response, LPS, Tregs
pMAPKAPK2 response, IL, Bcells
IkB response, IL, pDCs
prpS6 response, IL, $CD45RA^{+}$Tregs
pCREB response, IL, ncMCs
pNFkB response, IL, $CD56^{+}CD16^{-}$NKcells
IkB response, IL, $CD45RA^{-}$Tregs
prpS6 response, IFNα, Neutrophils
pERK response, IFNα, $Tbet^{+}CD8^{+}Tcells_{mem}$
pERK response, IFNα, $CD8^{+}Tcells_{mem}$
pNFkB response, IFNα, $CD56^{+}CD16^{-}$NKcells

Table S4. Reduced csEN model components.

csEN model components	Relative importance (-log(pvalue)*EN coefficient	Communities
**Endogenous pSTAT5, $CD4^{+}Tcells_{naive}$**	**11.70**	**7**
**pSTAT5 response, IL, Neutrophils**	**10.34**	**7**
**Endogenous, pSTAT5, $CD8^{+}$Tcells**	**6.90**	**7**
**pCREB response, LPS, $CD7^{+}$Nkcells**	**4.34**	**18**
**pCREB response, LPS, $CD56^{+}CD16^{-}$NKcells**	**3.79**	**18**
**pSTAT5 response, IFNα, $CD4^{+}$Tcells**	**3.23**	**14**
**pNFkB response, mDCs**	**3.15**	**2**
**pSTAT1 response, IFNα, $CD16^{+}CD56^{-}$NKcells**	**3.02**	**16**
prpS6 response, LPS, $CD56^{+}CD16^{-}$NKcells	3.01	19
IkB response, LPS, $CD45RA^{-}$Tregs	2.65	6
pSTAT5 response, IFNα, $Tbet^{+}CD8^{+}Tcells_{mem}$	2.04	1
Endogenous IkB, $CD8^{+}Tcells_{naive}$	2.01	19
pERK response, LPS, M-MDSCs	1.53	2
pERK response, IL, mDCs	1.53	8
IkB response, LPS, mDCs	1.51	19
$TCRgd^{+}$Tcells	1.18	19
pMAPKAPK2 response, LPS, mDCs	1.03	8
Endogenous prpS6, $TCRgd^{+}$Tcells	0.96	19
Endogenous, IkB, $CD8^{+}$Tcells	0.70	15
Endogenous, pSTAT5, pDCs	0.62	1
IkB response, LPS, $CD56^{+}CD16^{-}$Nkcells	0.58	6
pNFkB response, LPS, $CD56^{+}CD16^{-}$NKcells	0.50	5
pSTAT1 response, IFNα, mDCs	0.49	16
pSTAT3 response, IL, M-MDSCs	0.37	14
$CD8^{+}Tcells_{naive}$	0.35	19
